# Editorial: Advances on genomics and genetics of horticultural crops and their contribution to breeding efforts

**DOI:** 10.3389/fpls.2023.1186982

**Published:** 2023-03-31

**Authors:** Aliki Xanthopoulou, Eleni Tani, Christos Bazakos

**Affiliations:** ^1^ Institute of Plant Breeding and Genetic Resources, ELGO-DIMITRA, Thessaloniki, Greece; ^2^ Joint Laboratory of Horticulture, ELGO-DIMITRA, Thessaloniki, Greece; ^3^ Laboratory of Plant Breeding and Biometry, Agricultural University of Athens, Athens, Greece; ^4^ Max Planck Institute for Plant Breeding Research, Department of Comparative Development and Genetics, Cologne, Germany

**Keywords:** omics approaches, phylogenetics, breeding, quantitative genetics, bioinformatics

## 1 Introduction

Horticultural crops are an excellent source of vitamins, antioxidants, and fibers that play an important role in human health (Rajani and Joshi, 2017). The discovery and development of modern genomic tools, along with the use of comparative and functional genomics, have led to significant innovations with multiple applications in horticultural crops breeding (Singh et al., 2021). The present Research Topic aims to provide a collection of manuscripts with novel results on genomics and genetics of horticultural crops that potentially will contribute to the breeding efforts in the light of climate crisis. Jo et al. have published an article showcasing the assembly of the chloroplast genome sequence for *Vicia bungei* and they were able to unveil the evolutionary relationships present within the Vicia genus. Chen et al. studied the sequence characteristics, and they performed the phylogenetic analysis of 72 *Artemisia argyi* varieties chloroplast genome. Rau et al. determined the clonal population structure of a worldwide collection of globe artichokes providing crucial information for future genome-wide association mapping approaches. Choi et al. conducted a genome-wide re-annotation of the bZIP gene family in nine Solanaceae species as well as *Arabidopsis thaliana*, leading to a better comprehension of their roles within Solanaceae plants. Lu et al. investigated the roles of miR395 in cucumber fruit expansion and response to abiotic stresses. Finally, but no less importantly, Song et al. conducted a transcriptomic analysis of *Toona c*iliata stems in response to *Hypsipyla robusta* Moore. Through this analysis, they were able to gain insights into the molecular mechanisms responsible for the resistance of *T.ciliata* to *H.robusta* Moore.

## 2 Phylogenetic analyses using chloroplast and nuclear genome variation

*Artemisia argyi* Levl. et Van is a species belonging to Asteraceae family that contains a plethora of active ingredients with pharmaceutical utilities such as flavonoids, phenolic acids, volatile components, etc (Mei et al., 2016). Chen et al. collected 72 genotypes of *A.argyi* from 47 representative regions and their chloroplast (cp) genomes were systematically analyzed to detect a total of 196 polymorphic sites. Based on the phylogenetic tree, using 43 protein-coding genes that were found in all 67 Asteraceae species studied, *A.argyi* is closely related to *A.lactiflora* and *A.Montana*. Moreover, it appeared that Anthemidae Cass species had a closer relationship with some Astereae Cass species, compared to Senecioneae Cass species. These results assist in the identification and understanding of the evolutionary history of the family Asteraceae.

*Vicia L.* is comprised of 180–210 species and is a highly economically important genus due to its wide use as green manure, cover, forage, and honey crops (Montemurro et al., 2013). Jo et al. assembled the complete chloroplast genome of *Vicia bungei* and they have found 45 chloroplast genes to be involved in photosynthesis. The phylogenetic analysis of 20 Vicia spp. accessions clearly differentiated the accessions according to their genotype and clustered them into seven major groups. The cpSSR markers developed in *V.bungei* classified efficiently Vicia species genotypes and could be the springboard for advanced systematic breeding as well as charting the direction of conservation strategies.

Globe artichoke is a perennial open-pollinated crop (Pécaut, 1993) with many different clonal varietal groups adapted to different local environments. Rau et al. analysed a total of 110 globe artichoke accessions, representing most of the varieties cultivated worldwide, using simple sequence repeat (SSR) analysis and phenotypic characterization. The analyzed population clustered into two main genetic groups in agreement with Elia and Miccolis (1996). The observed low population size and strong bottleneck effects, indicate a long history of clonal propagation and selection during the evolution of the domesticated gene pool of globe artichoke. The comparison between molecular and phenotypic population structures revealed that harvest time, plant architecture, leaf spininess, head morphology and the number of heads per plant were the main pillars of selection during the evolution of the cultivated germplasm.

## 3 (A)Biotic stress related transcriptomic analyses

Playing important roles in plant growth, development, and biotic/abiotic stress responses, the bZIP gene family represents one of the largest transcription factor families (Baena-Gonzalez et al., 2007). Choi et al. focused on the re-annotation and comparative analysis of bZIP genes in nine Solanaceae species, as well as *A. thaliana*. By analyzing the gene ontology (GO) enrichment data, they identified that bZIP genes have diverse functions in pepper (*C.annuum*) and tomato (*S.lycopersicum*) under abiotic stress conditions. These results provide comprehensive information regarding the structure, expression, and functions of bZIP genes in Solanaceae.

*Toona ciliata*, is highly valued as a material for high-end furniture export due to its economic significance (Song et al., 2020). However, the leaves of *T. ciliata* are a preferred site for *Hypsipyla robusta* Moore (HRM) adult worms to lay eggs, leading to damage to the stems and apical buds from HRM larvae feeding on them. Song et al. discovered that there is no natural insect-resistant *T.ciliata* provenance, highlighting the importance of understanding the induced defense mechanism and relevant genes and pathways of endogenous insect resistance. Through their study aimed to clarify the molecular mechanism of *T.ciliata*’s response to HRM, providing valuable insights into the molecular mechanisms underlying its resistance to HRM.

Cucumber (*Cucumis sativus* L.) is one of the most important horticultural crops (Food and Agriculture Organization of the United Nations, 2021). It is highly important to elucidate the molecular mechanisms of cucumber fruit expansion to protect it from adverse conditions for higher yield and quality. Lu et al. recognized that miR395 is one of the highly conserved miRNAs and participates in plant metabolism *via* regulating downstream target genes (Yuan et al., 2016). To comprehend their roles in cucumber fruit expansion and response to abiotic stresses they identified and characterized four Csa-miR395s and eight corresponding target genes through bioinformatics analysis, revealing the chromosomal location of Csa-miR395s miRNAs and the genetic relationships between CsamiR395a/b/c and Csa-miR395d miRNAs. Promoter analysis of Csa-MIR395s identified various kinds of cis-acting elements, suggesting that Csa-miR395s might play a key role in regulating cucumber growth and development. Finally, data collected from qRT-PCR, RNA-seq, and bioinformatic analysis suggested that Csa-miR395a/b/c targeting Csa2G215520 and Csa1G502860 might affect cucumber fruit expansion by altering sulfur metabolism.

## Author contributions

All authors listed have made a substantial, direct, and intellectual contribution to the work and approved it for publication.
